# Use of Autotransfusion following Percutaneous Thrombectomy for Cardiogenic Shock Due to Pulmonary Embolism in a Single Session—A Case Report

**DOI:** 10.3390/diagnostics13081392

**Published:** 2023-04-11

**Authors:** Franz Haertel, Laura Baez, Marcus Franz, Jurgen Bogoviku, Friederike Klein, Gudrun Dannberg, P. Christian Schulze, Sven Möbius-Winkler

**Affiliations:** Department of Internal Medicine I, Division of Cardiology, University Hospital Jena, Am Klinikum 1, 07743 Jena, Germany

**Keywords:** pulmonary embolism, shock, acute heart failure, percutaneous mechanical thrombectomy, autotransfusion

## Abstract

A 64-year-old male patient was admitted to the catheterization laboratory with a suspected myocardial infarction and in cardiogenic shock. Upon further investigation, a massive bilateral pulmonary embolism with signs of right heart dysfunction was discovered, leading to a decision to perform a direct interventional treatment with a thrombectomy device for thrombus aspiration. The procedure was successful in removing almost the entirety of the thrombotic material from the pulmonary arteries. The patient’s hemodynamics stabilized and oxygenation improved instantly. The procedure required a total of 18 aspiration cycles. Each aspiration contained approx. 60 mL blood amounting to a total of approx. 1080 mL of blood. During the procedure, a mechanical blood salvage system was used to resupply 50% of the blood via autotransfusion that would otherwise have been lost. The patient was transferred to the intensive care unit for post-interventional care and monitoring. A CT angiography of the pulmonary arteries after the procedure confirmed the presence of only minor residual thrombotic material. The patient’s clinical, ECG, echocardiographic, and laboratory parameters returned to normal or near normal ranges. The patient was discharged shortly after in stable conditions on oral anticoagulation.

## 1. Introduction

Pulmonary embolism (PE) is a cardiovascular disorder characterized by the obstruction of pulmonary arteries by emboli, which are typically thrombi originating from the deep venous system of the lower extremities or pelvis. This obstruction can lead to impaired oxygenation of the affected lung tissue, manifesting clinically as chest pain, dyspnea, and tachypnea. In severe cases, PE can lead to cardiogenic shock if the obstruction of the blood flow to the lung by the emboli becomes critical. Immediate emboli-removal techniques such as percutaneous mechanical thrombectomy (PMT) may be considered in an effort to directly reverse shock as opposed to the application of systemically acting thrombolytic agents that might entail relevant bleeding complications. With the increasing availability of specific devices for such an intervention, this technique must be given special consideration.

## 2. Case Report

**Figure 1:**
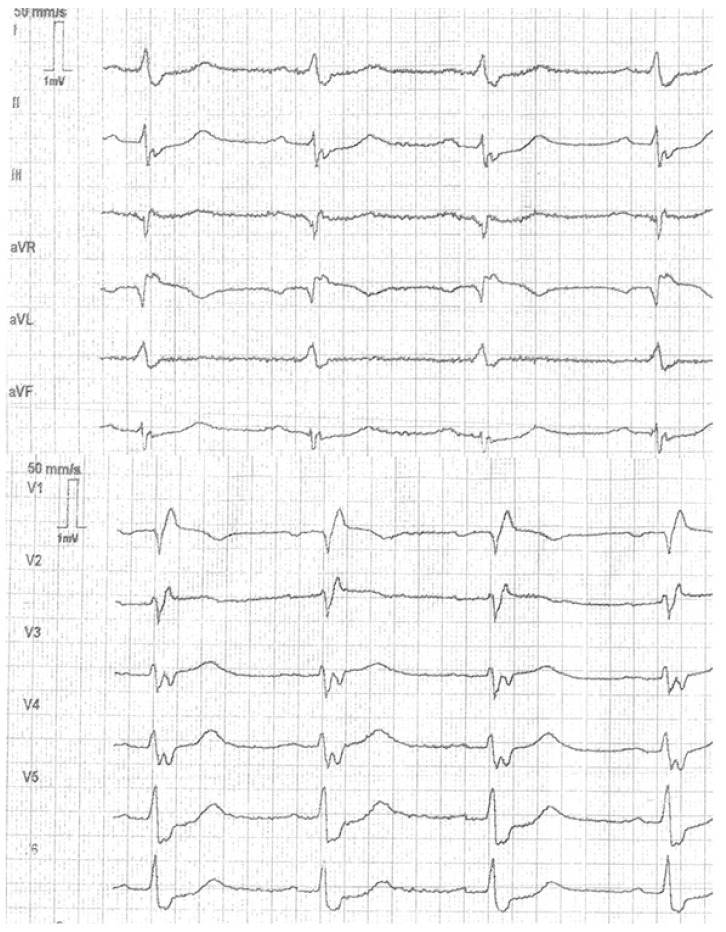
A 12-lead surface ECG showing ST segment elevations in aVR, V1 and V2.

A 64-year-old male patient (height: 186 cm; weight: 95 kg, body mass index: 27.5 kg/m^2^) was directly brought into our catheterization laboratory on the suspicion of a heart attack with the chief complaints of increasing shortness of breath and a short-lasting chest pain. A 12-lead surface ECG showed ST segment elevations in aVR, V1 and V2 as well as a right bundle branch block (Figure 1), so a cardiogenic shock due to myocardial infarction (STEMI) was primarily assumed [1]. At the same time, the laboratory and clinical findings indicated relevant abnormalities (Table 1).

**Figure 2:**
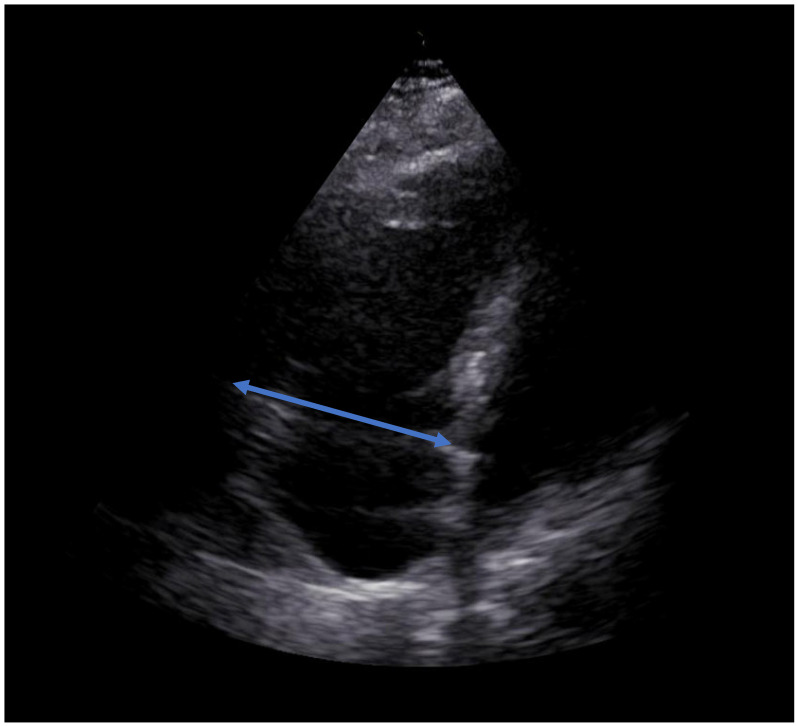
Baseline transthoracic echocardiogram showing a distended right ventricle with a right ventricular end-diastolic diameter (RVEDD) of 6.49 cm (arrow).

**Figure 3:**
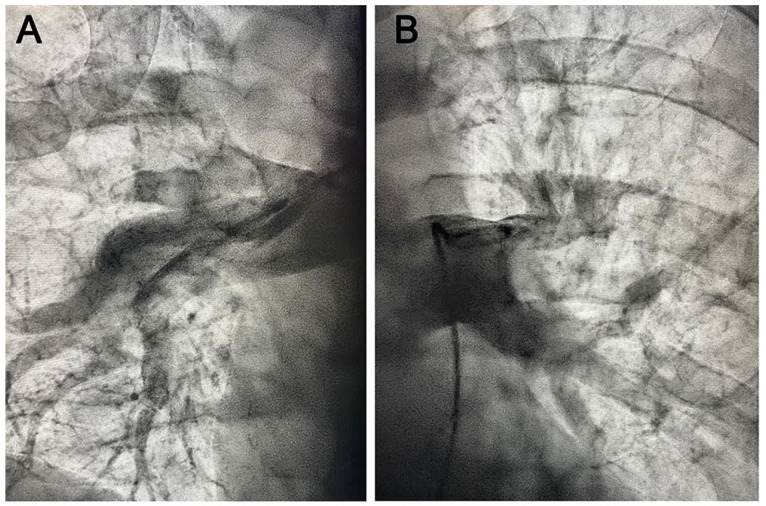

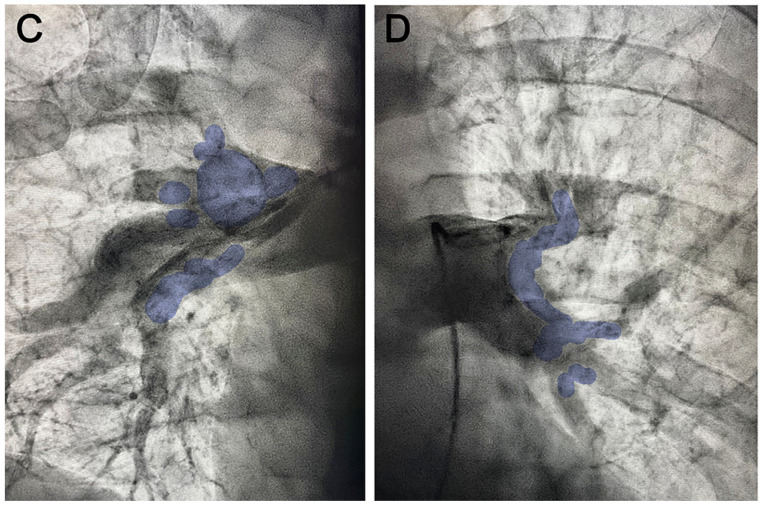
Right (**A**) and left (**B**) pulmonary artery angiography showing bilateral embolisms (colored markings in (**C**,**D**).

A relevant coronary artery disease was ruled out. The patient’s shock was aggravated with an elevated arterial lactate of 3.5 mmol/L, requiring an inotropic therapy (Table 1) and volume substitution (400 mL/h) to maintain a mean blood pressure of 60–70 mmHg. Due to the findings of the TTE showing primarily a right heart enlargement and impaired right ventricular function (Table 1, Figure 2), it was decided to perform an additional right heart catheterization with pulmonary angiography. A massive bilateral embolism was found (Figure 3), with a pulmonary artery pressure (PAP) of 44 mmHg systolic (sPAP), 23 mmHg diastolic (dPAP), and 29 mmHg mean (mPAP). On account of a hemodynamically relevant pulmonary embolism with a critical pulmonary perfusion deficit and clinically unresolved cardiogenic shock, arterial puncture and unknown patients’ status (e.g., undetected malignancies), it was deemed necessary to thoroughly evaluate these circumstances. After careful consideration, it was ultimately decided against pursuing systemic thrombolysis as this method is not reasonably free of danger following arterial access during coronary angiography. A further decision was made favoring immediate interventional treatment using the FlowTriever^®^ device (INARI Medical^®^, Irvine, CA, USA) for direct thrombus aspiration (percutaneous mechanical thrombectomy (PMT)).

**Figure 4:**
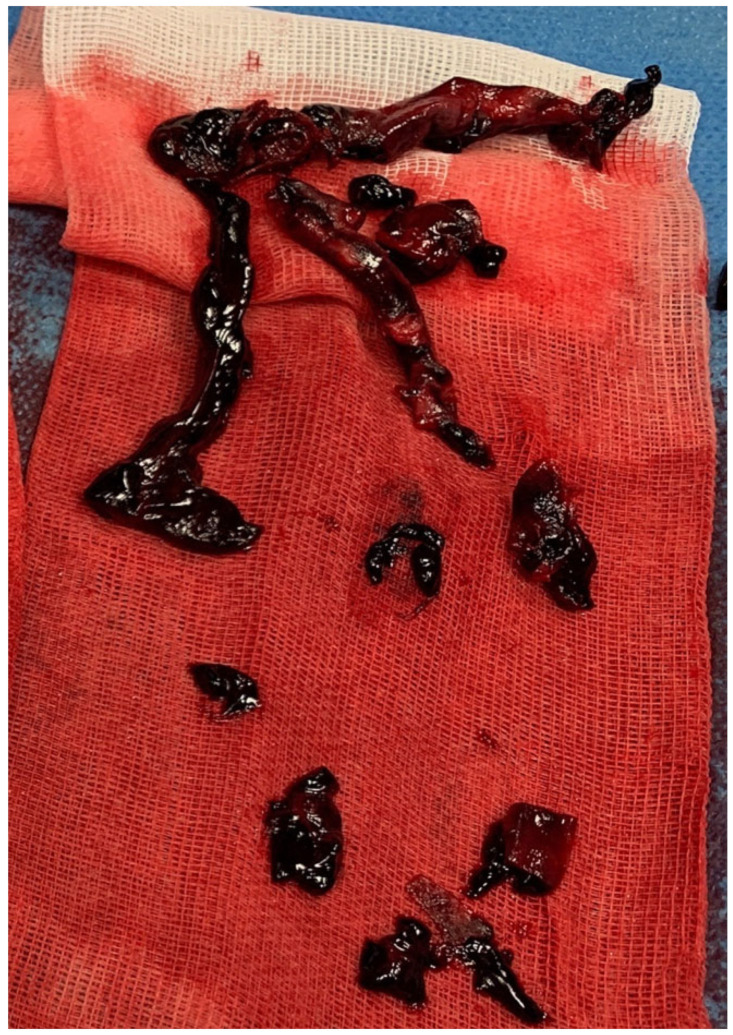
Fresh and organized thrombotic material retrieved via direct percutaneous, pulmonary thrombus aspiration.

**Figure 5:**
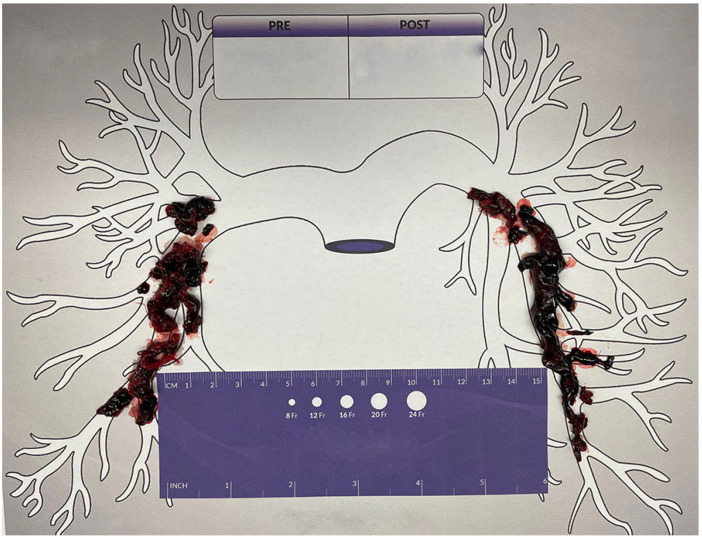
Retrieved thrombotic material and origin within the pulmonary artery system.

The left pulmonary artery was targeted first. After the fourth aspiration cycle, the hemodynamic circulation shifted towards more and more hypertensive blood pressure values (systolic pressure >150 mmHg) and the volume rate of the inotropic therapy and fluid substitution could be dramatically reduced. The retrieved thrombus material was partly fresh and partly already organized. The patient was increasingly relived from shortness of breath. After 30 min, a total of 18 aspiration cycles removed most of the thrombotic material from both pulmonary arteries (Figure 4 and Figure 5). Each aspiration contained 40–60 mL of blood amounting to a total of 720–1080 mL of blood. During the intervention, an activated clotting time (ACT) of over 300 s was maintained via intravenous unfractioned heparin. Already after the procedure and still inside the catheterization laboratory, all inotropes and volume resuscitations could be stopped. Arterial blood gas analysis showed a normalized arterial lactate of 1.5 mmol/L. Vital signs improved as well (Table 1) and O_2_ supplementation could be de-escalated to a continuous flow delivery via a nasal canula. Pulmonary artery catheter (PAC) hemodynamics confirmed an improved pulmonary circulation (sPAP/dPAP/mPAP: 25/10/16 mmHg). During the entire procedure, the patient was adequately awake (Glasgow Coma Scale (GCS) of 15).

**Figure 6:**
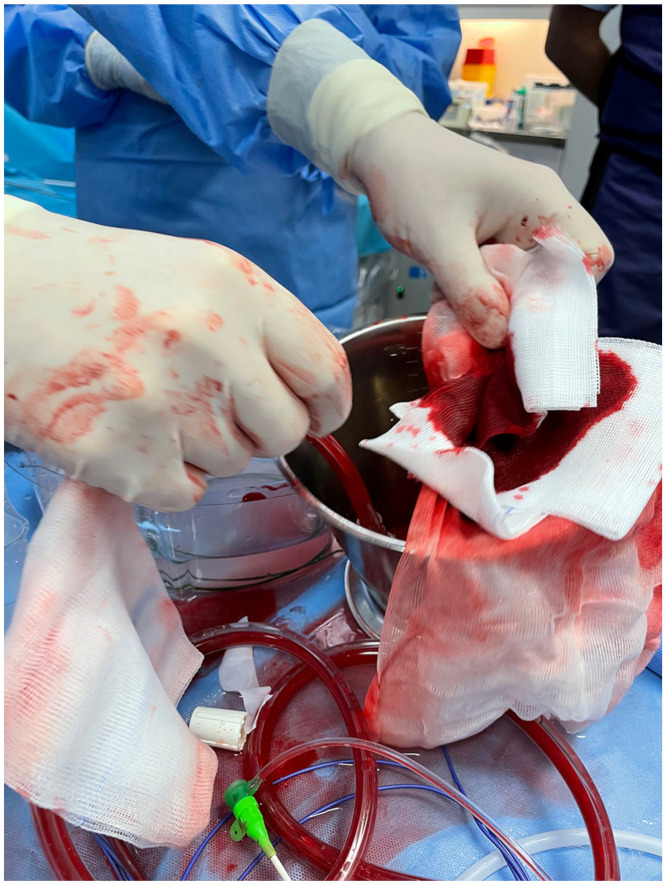
The removed blood during the aspiration procedure is filtered through gauze and collected in a cup. The collected fluid is then transferred into the Cell Saver^®^ to be processed for autotransfusion.

**Figure 7:**
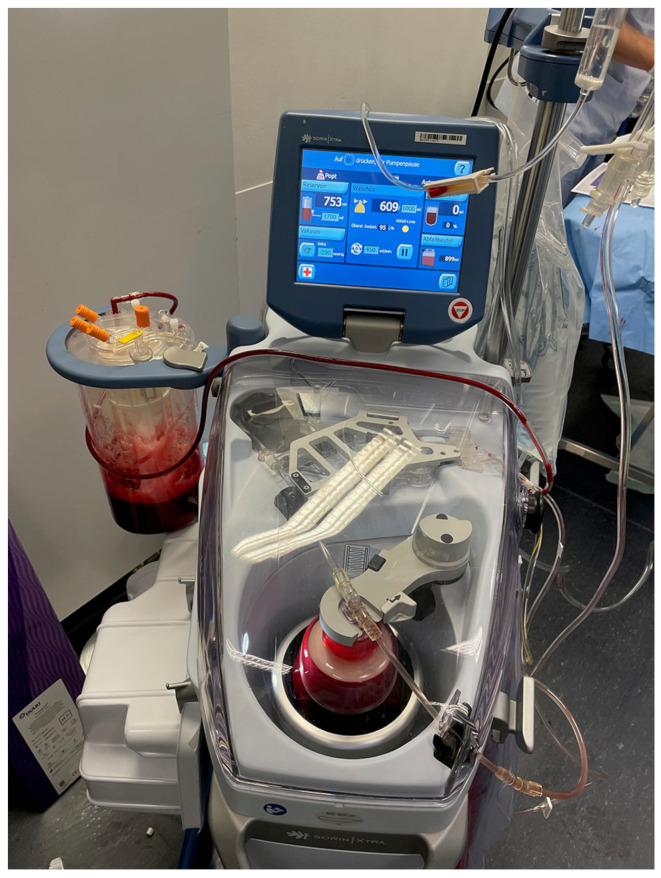
Cell Saver^®^ in operation processing the retrieved blood for autotransfusion.

The aspirated blood that would have been otherwise discarded was salvaged during the procedure using a mechanical autotransfusion system (Cell Saver^®^, Haemonetics^®^, Boston, MA, USA, Figure 6 and Figure 7). The blood underwent centrifugation, resulting in the enrichment of red blood cells to a hematocrit level of 50%. Approx. 600 mL of blood concentrate could be reinfused directly after the procedure. No relevant abnormalities were detected after the entire procedures in the levels of hemoglobin, hematocrit, or platelets (Table 1).

**Figure 8:**
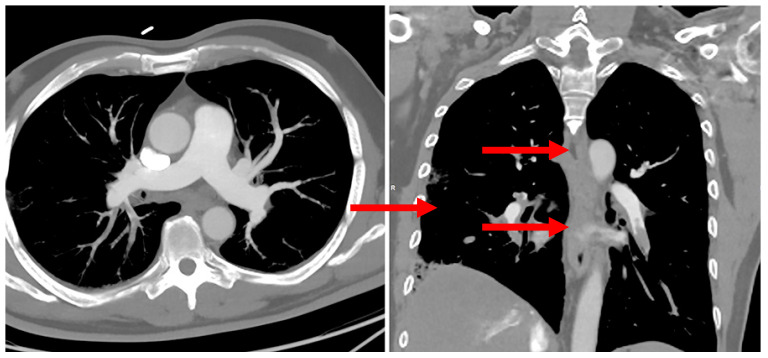
**Left** side: Transverse view of both pulmonary arteries after the thrombectomy showing no central thrombotic material. **Right** side: Coronal view showing only minor residual subsegmental thrombotic material (arrows).

The patient was transferred in stable hemodynamic conditions (Table 1) to our intensive care unit (ICU) for postinterventional monitoring and continuation of intravenous unfractioned heparin application with a target aPPT of 60–70 s. A CT-angiography of the pulmonary circulation after the procedure verified only minor residual subsegmental thrombotic material (Figure 8). A large thrombus was detected within the left popliteal vein as the likely origin of the embolism.

**Figure 9:**
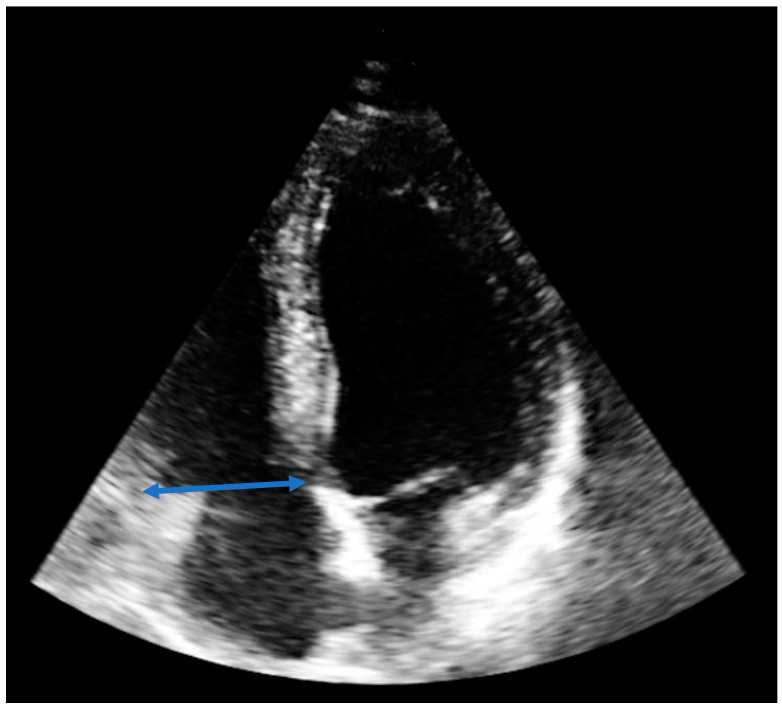
Transthoracic echocardiogram before discharge showing a normalized right ventricle with a right ventricular end-diastolic diameter (RVEDD) of 3.7 cm (arrow).

**Figure 10:**
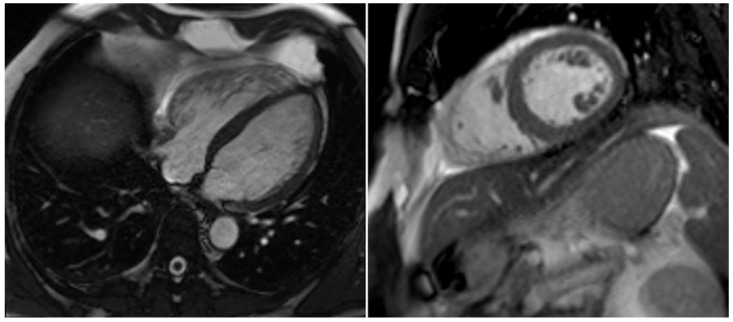
Four chamber view (**left** side) and short axis view (**right** side) of a cardiac magnetic resonance imaging (MRI) four weeks after the procedure. Right ventricular failure could be excluded; however, prominent trabeculation was present.

Relevant clinical, ECG, echocardiographic (Figure 9) and laboratory parameters returned to or near normal ranges (Table 1) until discharge. A final 6-min walk test (6MWD) showed no pulmonary limitations or oxygenation impairments (distance of 420 m (pO_2_: 78.09 mmHg)). The patient’s status during an outpatient appointment remained uneventful, and a follow-up cardiac MRI showed no signs of right heart failure or dilation, but merely prominent trabeculation (Figure 10). This suggests that the patient has not experienced any complications from the treatment and is on a positive trajectory for full recovery.

## 3. Discussion

Globally, venous thromboembolism (VTE), particularly PE, is the third leading cause of cardiovascular deaths, after myocardial infarction and cerebrovascular diseases [2]. In the United States, PE results in around 150,000–250,000 hospital admissions and 60,000–100,000 deaths annually [3].

There are several treatment modalities for massive PE, including systemic thrombolysis, catheter-directed thrombolysis (e.g., ECOS^®^, Boston Scientific^®^, Marlborough, MA, USA), surgical embolectomy and PMT [4,5].

While anticoagulation therapy has traditionally been the primary treatment modality for most cases of PE [6], more severe cases of larger emboli that involve hemodynamic impairment may require a prompt, more aggressive approach. Additionally, thrombolytic therapy may not be appropriate for patients with certain conditions, such as recent head trauma, stroke, malignancies or pregnancy.

Determining the hemodynamic status of patients with PE is crucial for accurate diagnosis, effective treatment, and proper prognosis. In addition, the ratio of the right ventricle to left ventricle diameter, as determined by our transthoracic echocardiography findings, which showed clear dilation of the right ventricle, is the strongest predictor of short-term mortality and negative outcomes in cases of severe PE and can guide treatment decisions [7].

Therefore, for unstable patients who cannot safely undergo systemic fibrinolysis or who have failed thrombolysis, PMT may be a viable alternative as was the case for our patient. PMT has been shown to provide rapid hemodynamic benefits, minimize the risk of bleeding, and reduce the duration of hospitalization and intensive care unit stay, leading to cost savings compared to thrombolysis [8,9,10] as we could prove with our case.

Currently, there are no clear guidelines on when to use PMT in the treatment of massive/submassive PE. The European Society of Cardiology and European Respiratory Society recommend using PMT in patients with high-risk PE if thrombolysis is not an option or not effective [11]. In cases of severe circulatory collapse or cardiac arrest, catheter-directed treatment combined with extracorporeal membrane oxygenation (ECMO) may be considered as an option [11].

The FLARE trial by Tu et al. [10] evaluated the safety and effectiveness of percutaneous mechanical thrombectomy using the FlowTriever^®^ System in patients with acute intermediate-risk pulmonary embolism. Patients with symptomatic, computed tomography-documented PE and right ventricular (RV)/left ventricular (LV) ratios ≥0.9 were eligible for enrollment. The primary effectiveness endpoint was core laboratory-assessed change in the RV/LV ratio, and the primary safety endpoint was device-related death, major bleeding, treatment-related clinical deterioration, pulmonary vascular injury, or cardiac injury within 48 h of thrombectomy. A total of 106 patients were treated with the FlowTriever^®^ System, with a mean procedural time of 94 min and mean ICU stay of 1.5 days. At 48 h post-procedure, the average RV/LV ratio reduction was 0.38 (25.1%). The study found that a percutaneous mechanical thrombectomy with the FlowTriever^®^ System appears safe and effective in patients with acute intermediate-risk PE, with significant improvement in the RV/LV ratio and minimal major bleeding.

Mathbout et al. [12] presented a case series and literature review of three intermediate-high risk submassive PE patients treated with the FlowTriever^®^ system. The authors reported a significant reduction in pulmonary artery systolic pressure and improvement in right ventricular function in all patients that can be related to our experiences with the presented case. Overall, the findings of this case series suggest that the mechanical thrombectomy system may be a safe and effective treatment option for intermediate-high risk submassive PE patients.

However, due to the number of aspirations necessary until a sufficient portion of thrombotic material has been removed, relevant blood losses can be iatrogenically inflicted to the patient. In addition to obstructive shock coming from PE itself, a patient can further sustain hypovolemic shock. It is particularly concerning in situations of shock, where an intravascular volume depletion and thus loss of valuable blood components potentially aggravates the downward shock spiral. It is possible to estimate the blood volume of an adult: The Lemmens–Bernstein–Brodsky formula allows for the estimation of an adult’s blood volume based on their weight and height [13]. This formula provides a rough approximation, as the actual blood volume may vary due to personal factors and health conditions. In the presented case, this results in a blood volume of 6023 mL (≈6 L) of our presented patient. A different source states that the total circulating blood volume accounts for approximately 7% of the total body weight [14], which equals approximately 6.7 L in our patient. During the treatment of our patient, an approximate blood loss of 1100 mL was incurred. This equates to approximately 18% of the total blood volume, assuming a total blood volume of 6 L. Losses of less than 15% of the total blood volume typically result in a slight elevation of heart rate and negligible alterations in arterial pressure [14]. Conversely, blood losses between 15% and 40% result in a decline in mean arterial and pulse pressures and an increase in heart rate, with the degree of these changes proportional to the extent of blood loss [14].

## 4. Autotransfusion Procedure

The autotransfusion process begins by aspirating fluid from the operating/interventional field using a special double lumen suction tubing, mixed with an anticoagulant solution, and filtered in a sterile reservoir with a capacity of 2–3 L [15]. After filtration, the red blood cells are concentrated, washed, and re-infused as red blood cells suspended in normal saline with a hematocrit of around 60%. Platelets and plasma, including both desired and undesired components, are removed [15].

Autotransfusion, which involves the use of a person’s own blood for transfusion, is typically used in cases with substantial blood loss, typically exceeding 1000 mL [15,16]. It may be particularly useful in situations where the patient has a rare blood group, a risk of infectious disease transmission, supply of homologous blood is limited, or there are other medical and non-medical reasons, such as religious aspects, why the use of homologous blood is not recommended [15]. Autotransfusion may also be recommended in cases of urgent blood need, unavailability or inability to provide cross-matched blood, or patient refusal to receive cross-matched blood [17]. Autotransfusion can be used both during and after interventions [15]. It is typically utilized in major surgical procedures where a significant blood loss is anticipated [15].

Autotransfusion has several benefits compared to the use of allogeneic (donated) blood [15]. It can provide cells with higher levels of 2,3-diphosphoglycerate (DPG), which can improve oxygen delivery to tissues [18]. The blood is normothermic and has a relatively normal pH, which can help to preserve the function of the transfused cells [18]. There is also a lower risk of infectious diseases being transmitted through autotransfused blood [15]. In addition, the transfused cells may be functionally superior to stored allogeneic blood cells [19]. Autotransfused blood has a lower potassium level compared to stored allogeneic blood, which can be beneficial in certain situations [15,20]. Autotransfusion is quickly available and may reduce the need for allogeneic red cell transfusion during certain surgeries, such as adult elective cardiac and orthopedic surgery [15].

Autotransfusion has some potential disadvantages to consider. During the process of producing an autotransfusion, certain substances and components are lost or need to be removed, including plasma, platelets, white cells, anticoagulant solution, plasma free hemoglobin, cellular stroma, activated clotting factors, intracellular enzymes, potassium, and plasma-bound antibiotics [15,20]. This may limit the usefulness of autotransfusion in certain situations.

Known problems associated with autotransfusion such as low total protein levels causing peripheral or pulmonary edema or coagulation problems did not occur in our patient. An in vitro study has shown that the coagulation factors can withstand significant dilution without impairing the clotting time [21]. The Cell Saver^®^ device was found to be a highly effective, dependable, and safe method for autotransfusing salvaged blood during cardiovascular surgeries [21]. Complications can be avoided in general by using sterile techniques during autotransfusion procedures and limiting the amount of reinfused blood to less than 3000 mL [15,20].

Contamination is a significant contraindication for autotransfusion. If the source of contamination is infectious, the wound site must be carefully examined for any signs of contamination before using the collected blood [20,22]. If there is any uncertainty regarding the sterility of the collected blood, it is recommended to use cross-matched blood instead [20,22]. On the other hand, if the source of contamination is non-infectious, it is best to avoid using blood that may have been mixed with a sterile solution, such as iodine, sterile water, alcohol, irrigation solutions, or chlorhexidine [20,22]. In cases where coagulopathy or disseminated intravascular coagulopathy (DIC) is present, specific blood component therapy is recommended instead of autotransfusion [23]. Furthermore, some studies suggest that autotransfusion should be avoided in cases of sickle cell disease and cesarean delivery, until further studies can confirm its safety and efficacy [20,24,25,26].

We present a novel case report of utilizing the Cell Saver^®^ for autotransfusion in a patient undergoing thrombectomy for pulmonary embolism using the FlowTriever^®^ Device. Our intervention successfully minimized blood loss in a critically ill patient experiencing cardiogenic shock, which represents a valuable contribution to the advancement of medical practice.

## 5. Conclusions

Performing a direct percutaneous thrombectomy even in cases of cardiogenic shock is technically possible, however it depends on the availability of local resources and the expertise of the medical staff. It could be demonstrated that such an approach leads to the immediate reversal of all shock signs and symptoms and uncompromised recovery of the patient. Relevant blood losses can be safely and effectively compensated in the same session during or after the procedure via the recycling of the aspirated blood using mechanical autotranfusion.

Learning points (1) Pulmonary mechanical thrombectomy can be an effective treatment option for patients with pulmonary embolisms who are experiencing hemodynamic instability or who are not responding to anticoagulation therapy. (2) Autotransfusion using the Cell Saver^®^ is a safe and reliable method for salvaging and reinfusing a patient’s own blood during pulmonary mechanical thrombectomy. (3) The combination of pulmonary mechanical thrombectomy and autotransfusion using the Cell Saver^®^ can minimize blood loss and potentially reduce the need for blood transfusions in critically ill patients undergoing this procedure.

## Figures and Tables

**Table 1:** Selection of pre- and post-interventional patient characteristics following percutaneous thrombectomy of bilateral pulmonary embolism and autotransfusion.

	Preinterventional	Immediately after the Procedure	At Discharge 6 Days after the Procedure
Clinical data			
Dyspnea [NYHA]	IV	II–III	0
Arterial blood pressure [systolic/diastolic/mean, mmHg]	73/44/70	103/70/84	122/61/81
Heart rate [beats/minute]	100	81	64
SpO_2_ [%]	90	95	97
Oxygen supplementation [liters/minute]	10	3	0
Respiratory rate [breaths/minute]	29	16	12–14
Echocardiographic data			
RVEDD [mm]	65	49	37
TAPSE [mm]	12	16	21
TASV [cm/s]	8	12	17
sPAP [mmHg]	55	15	14
LVEF [%]	43	54	56
D- Sign	Yes	No	No
McConnell‘s sign	Yes	Yes	No
Systolic notching of PWD over pulmonary valve	Yes	Slightly	No
PAC hemodynamics			
PAP [systolic/diastolic/mean, mmHg]	44/23/29	25/10/16	Not available
Laboratory data			
D-Dimer [µg/L]	41,531	Not available	Not available
Troponin [pg/mL]	75.20	505	21.8
Myoglobin [µg/L]	80	36	Not available
NT-Pro-BNP [pg/mL]	207	1062	<50
Arterial lactate [mmol/L]	3.5	1.5	Not available
Hb [mmol/l]	10	8.2	9.7
Thrombocytes [Gpt/l]	175	142	315
Hematocrit [%]	47	38	42
Therapy data			
Noradrenalin [µg/kg/min]	0.47	0	0
Dobutamine [µg/kg/min]	8.3	0	0
Unfractionated intravenous heparin	Yes	Yes	No
NOAC	No	No	Yes (Apixaban)

Hb = Hemoglobin; LVEF = Left ventricular ejection fraction; NOAC = New oral anticoagulants, NT-pro-BNP = N-terminal prohormone of brain natriuretic peptide; NYHA = New York Heart Association; PAC = Pulmonary artery catheter; PAP = Pulmonary artery pressure; PWD = Pulse wave doppler; RVEDD = Right ventricular enddiastolic diameter; TAPSE= Tricuspid annular plane systolic excursion; TASV = Tricuspid annular systolic velocity.

## Data Availability

This is a case report. All data are included in the manuscript.
